# Supplementary material for: Construct validity and internal consistency of the Breast Inflammatory Symptom Severity Index in lactating mothers with inflammatory breast conditions
Source: PeerJ. 2021 Nov 16;9:e12439. doi: 10.7717/peerj.12439 (PMC8603819; doi:10.7717/peerj.12439)
Supplement: Supplemental Information 1 [file peerj-09-12439-s001.docx]

## **Table S1:**

## **Occupational socioeconomic status scale – modified version of Marks (2000)** (Marks et al. 2000)

| **Group 1:** Senior management in large business organisation government administration and defence and qualified professionals. |
| --- |
| - Senior executive/manager/department head in industry, commerce, media or other large organisation. - Public service manager (section head or above), regional director, health/education/police/fire services administrator. - Other administrator (school principal, faculty head/dean, library/museum/gallery director, research facility director). - Defence Forces commissioned officer. - Professionals generally have degree or higher qualifications and experience in applying this knowledge to design, develop or operate complex systems; identify, treat and advise on problems; and teach others. - Health, education, law, social welfare, engineering, science, computing professional. - Business (management consultant, business analyst, accountant, auditor, policy analyst, actuary, valuer). - Air/sea transport (aircraft/ship’s captain/officer/pilot, flight officer, flying instructor, air traffic controller). |
| **Group 2:** Other business managers, arts/media/sportspersons and associate professionals. |
| - Owner/manager of farm, construction, import/export, wholesale, manufacturing, transport, real estate business. - Specialist manager (finance/engineering/production/personnel/industrial relations/sales/marketing). - Financial services manager (bank branch manager, finance/investment/insurance broker, credit/loans officer). - Retail sales/services manager (shop, petrol station, restaurant, club, hotel/motel, cinema, theatre, and agency). - Arts/media/sports (musician, actor, dancer, painter, potter, sculptor, journalist, author, media presenter, photographer, designer, illustrator, proof reader, sportsman/woman/ coach, trainer, sports official). - Associate professionals generally have diploma/technical qualifications and support managers and professionals. - Health, education, law, social welfare, engineering, science, computing technician/associate professional. - Business/administration (recruitment/employment/industrial relations/training officer, marketing/advertising specialist, market research analyst, technical sales representative, retail buyer, office/project manager). - Defence Forces senior non-commissioned officer. |
| **Group 3:** Tradesmen/women, clerks and skilled office, sales and service staff. |
| - Tradesmen/women generally have completed a four-year trade certificate, usually by apprenticeship. All tradesmen/women are included in this group. - Clerks (bookkeeper, bank/PO clerk, statistical/actuarial clerk, accounting/claims/audit clerk, payroll clerk, recording/ registry/filing clerk, betting clerk, stores/inventory clerk, purchasing/order clerk, freight/transport/ shipping clerk, bond clerk, customs agent, customer services clerk, admissions clerk). - Skilled office, sales and service staff. - Office (secretary, personal assistant, desktop publishing operator, switchboard operator). - Sales (company sales representative, auctioneer, insurance agent/assessor/loss adjuster, market researcher). - Service (aged/disabled/refuge/child care worker, nanny, meter reader, parking inspector, postal worker, courier, travel agent, tour guide, flight attendant, fitness instructor, casino dealer/supervisor). |
| **Group 4:** Machine operators, hospitality staff, assistants, labourers and related workers. |
| - Drivers, mobile plant, production/processing machinery and other machinery operators. - Hospitality staff (hotel service supervisor, receptionist, waiter, bar attendant, kitchen hand, porter, and housekeeper). - Office assistants, sales assistants and other assistants. - Office (typist, word processing/data entry/business machine operator, receptionist, office assistant). - Sales (sales assistant, motor vehicle/caravan/parts salesperson, checkout operator, cashier, bus/train conductor, ticket seller, service station attendant, car rental desk staff, street vendor, telemarketer, shelf stacker). - Assistant/aide (trades’ assistant, school/teacher’s aide, dental assistant, veterinary nurse, nursing assistant, museum/ gallery attendant, usher, home helper, saloon assistant, animal attendant). - Labourer and related workers. - Defence forces ranks below senior NCO not included above. - Agriculture, horticulture, forestry, fishing, mining worker (farm overseer, shearer, wool/hide classer, farm hand, horse trainer, nurseryman, greenkeeper, gardener, tree surgeon, forestry/logging worker, miner, seafarer/fishing hand). - Other worker (labourer, factory hand, storeman, guard, cleaner, caretaker, laundry worker, trolley collector, car park attendant, crossing supervisor). |
| **Group 5:** Not currently in paid work |
| - Researcher/studying - Parental responsibilities/domestic manager |
| **Group 6:** Not reported |

## **References**

Marks GN, McMillan J, Jones FL, and Ainley J. 2000. The measurement of socioeconomic status for the reporting of nationally comparable outcomes of schooling. *Draft Report by the National Education Performance Monitoring Taskforce, Australian Council for Educational Research and Sociology Program, Research School of Social Sciences, Australian National University, www mceecdya edu au/verve/_resources/socioeconomicstatus_file pdf*.
